# What’s the Goal? Reconsidering the Importance of Higher-Order Cognition in Internalizing Psychopathology

**DOI:** 10.5334/cpsy.184

**Published:** 2026-11-13

**Authors:** Marta Migó, Michael T. Treadway

**Affiliations:** 1 Department of Psychology, Emory University, 30322, Atlanta, GA, United States; 2 Department of Psychiatry and Behavioral Sciences, Emory University School of Medicine, 30322, Atlanta, GA, United States

**Keywords:** goal selection, internalizing psychopathology, reinforcement learning, rumination, anhedonia

## Abstract

To date, the field of computational psychiatry has largely focused on testing for dysfunction within lower-level cognitive processes in psychopathology, such as biases related to attention, reinforcement learning and memory. While this work has yielded important insights, there is clear evidence from empirically validated psychotherapeutic interventions to suggest that these lower-level cognitive processes are often intact. Here, we argue that etiological and maintaining factors for internalizing disorders may be more appropriately situated in higher level cognitive processes such as goal-selection. We further suggest that future research should incorporate behavioral tasks and models that allow for participant-defined goals to reveal higher-level mechanistic targets. We believe that considering these higher-level deficits in goal-selection, possibly in tandem with low-level impairments, may augment ecological validity and clinical relevance.

## Introduction

Over the last decade, a major area of focus for research on the etiology of internalizing psychopathology has been the identification of biased information processing within low-level cognitive processes (e.g., LeMoult & Gotlib, 2019). This work has targeted processes such as biased attention, reinforcement learning and memory recall with the goal of developing generative models that can link symptomatic behavior to the under- or over-activation of specific cognitive systems, thereby helping to move psychiatric nosology beyond descriptive checklists and towards etiologically-grounded diagnostic criteria (Thomas & Sharp, 2019). To date, however, empirical evidence supporting the presence of clear deficits in these lower-level processes within clinical populations has often been inconclusive (Pike & Robinson, 2022). For instance, reinforcement-learning studies in major depressive disorder and anxiety-related disorders reveal heterogeneous and sometimes intact basic learning parameters rather than a consistent deficit profile (C. Chen et al., 2015; Moutoussis et al., 2018). Anxiety is further inconsistently associated with greater choice stochasticity (Pike & Robinson, 2022). Similarly, studies have reported conflicting effects of depressive symptoms on delay-discounting behavior (Mukherjee et al., 2023). This pattern of mixed findings raises important questions regarding the utility of many proposed low-level mechanisms.

Complementing these heterogeneous results from laboratory studies is the evidence from psychotherapy intervention studies, which suggest that low-level processes may be intact for some patients. For example, Behavioral Activation therapy works by encouraging individuals to engage in rewarding activities, thereby promoting the learning of new behavioral patterns that counteract avoidance, and ultimately improve mood through increased contact with positive reinforcement (Cuijpers et al., 2007; Kanter et al., 2015; Mazzucchelli et al., 2009). In other words, the effectiveness of this approach is partly dependent on the presence of an intact learning and decision-making system to aid in the development of new reinforcing behaviors and reach recovery. This raises the possibility that for at least some individuals, their symptoms result from deficits in higher order cognitive processes. Further, it has critical implications for understanding who may or may not benefit from these types of psychotherapies.

One such higher order process is goal selection, which refers to the cognitive process by which individuals evaluate, prioritize, and commit to specific objectives based on internal motivations, environmental demands, and anticipated outcomes (Verschure et al., 2014). Indeed, goal-selection is commonly discussed and targeted in psychotherapy settings, as it is consistently observed to be sub-optimal in these clinical populations (J. M. Dickson & Moberly, 2013; Weinberger et al., 2009). For instance, the Acceptance and Commitment Therapy framework encourages individuals with anxiety to select activities that will bring them closer to living in alignment with their long-term values, rather than activities that will decrease their levels of distress in the short-term (Hayes et al., 2006). Taken in conjunction with other recent research on the importance of goal selection (discussed below), this work suggests that goal-selection may be an overlooked cognitive mechanism underlying internalizing psychopathology.

Our claim is not that low-level cognitive processes never play a causal role in the development of internalizing psychopathology (indeed, see *Goal understanding, identification, and selection are crucial in therapeutic environments* for a discussion of effective psychotherapies that target low-level deficits). Rather, we suggest that this approach may fail to capture causal mechanisms for individuals who primarily struggle with higher-order processes. Moreover, it is also possible that what may have started as a low-level process deficit may have evolved to a goal-selection deficit by the time individuals reach a researcher’s testing room or a therapy session. Consider, for example, a learned attentional bias towards threatening stimuli, following a traumatic event. This attentional bias, in the short-term, may lead to an unpleasantly overactive sympathetic nervous system, and an exhausting tendency to remain hypervigilant. In the long-term, individuals may start to select activities that don’t trigger those unpleasant bodily reactions, effectively setting distress-avoidance goals in various aspects of their lives that may persist beyond the period of threat hypervigilance.

Accordingly, this article will aim to discuss the importance of moving towards research task paradigms that allow for a greater flexibility of goal-selection, hence, behaviors. We believe that studying differences in goal-selection in these populations may (1) explain previous inconsistent findings indicating some impairments in low-level cognition, (2) better align with clinical insights into these illnesses, and (3) help enhance ecological validity and the robustness of our conclusions. We draw from computational, cognitive, and clinical science to support our claim that studying differences in goal-selection may reveal core mechanisms underlying the development, maintenance, and/or progression of internalizing psychopathology.

## Goal-selection differences may impact learning and decision-making deficits linked to internalizing psychopathology

It is widely accepted that internalizing psychopathologies, such as depression and anxiety, impact the ways in which individuals perceive and interact with the world (Bishop & Gagne, 2018; Y. Chen et al., 2018). In particular, previous work has suggested that, in comparison to healthy controls, these populations are differentially sensitive to learning from feedback (Brown et al., 2021; Heller et al., 2018), are differentially sensitive to effort, rewards and punishments (Cavanagh et al., 2011; Mehrhof & Nord, 2025; Pulcu et al., 2024), show impairments in contingency adjustment (Gagne et al., 2020), and engage in non-normative patterns of foraging, exploration and stochastic choice in laboratory settings (Bishop & Gagne, 2018; Bustamante et al., 2024; Smith et al., 2022). Importantly, many computational models of behavior in internalizing psychopathology rely on the reinforcement learning (RL) framework (Pike & Robinson, 2022), partially because it was once implicitly believed that using a common computational framework across studies would enhance our ability to reconcile or contrast findings (Eckstein et al., 2021). However, we are now aware that differences in individuals’ goals (Frömer et al., 2025; Molinaro & Collins, 2023a) (both researcher and participant-generated goals) while completing a task can greatly impact task behavior and RL outputs. Indeed, goal-selection is pertinent to state representation development (Flesch et al., 2022; Mante et al., 2013), action perception (Rmus et al., 2023) and planning (Sharp & Eldar, 2024), and value assignment to rewards (De Martino & Cortese, 2023; Harlow, 1950; Keramati & Gutkin, 2014) –all fundamental components underlying RL. As such, inadequate consideration of goal-selection during an RL task can significantly confound the interpretation of classic RL outputs. In other words, failing to control for individual differences in goal-selection—particularly among those with internalizing psychopathology—may critically limit our ability to draw conclusions about the cognitive deficits underlying these conditions. Indeed, the failure to account for this higher-level process (goal-selection) could partially explain some of the inconsistencies that our field has produced (Pike & Robinson, 2022).

## What do individuals consider when selecting a goal?

There is a large extant literature on the dimensions that compose goal-selection that is beyond the scope of the present paper. Here, we focus on three key features that appear most relevant to goal-selection among individuals with various forms of internalizing psychopathology (see Figure 1).

**Figure 1:**
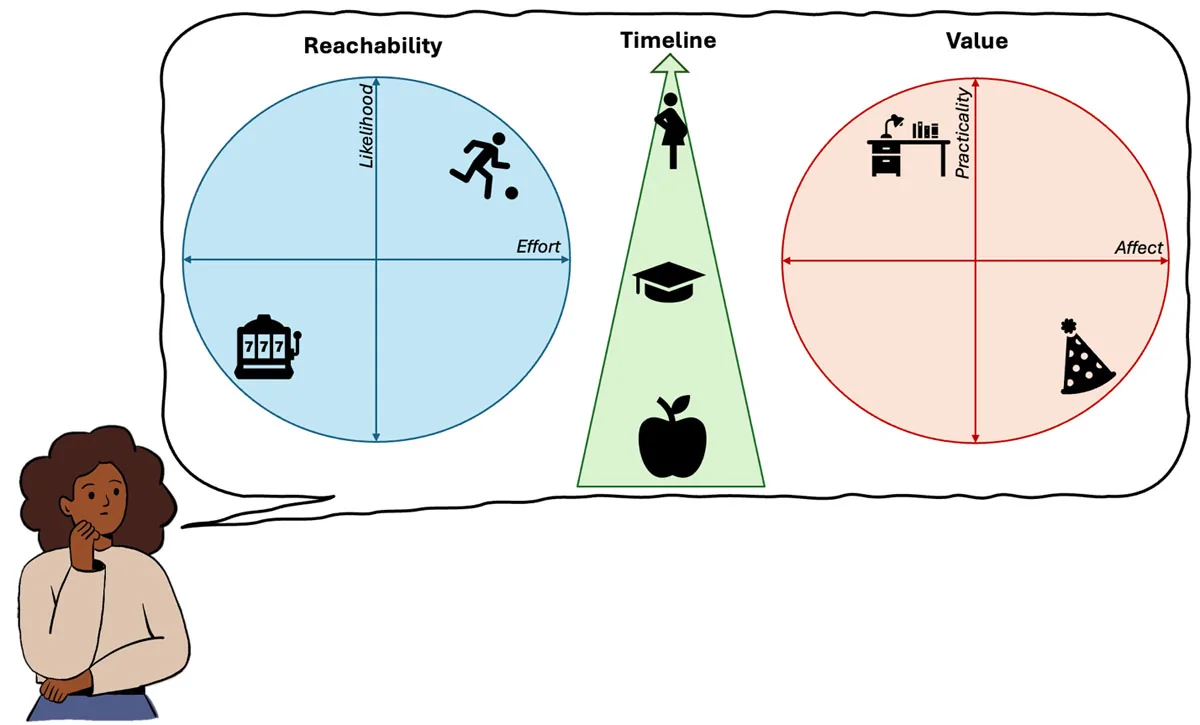
Visual of three possible contributors to goal selection: reachability, timeline, and value. Left: Reachability of a goal relies on the combination of a goal’s external likelihood and an individual’s effort towards it. Center: Timeline of a goal impacts its selection, planning, and execution. Right: Goal value may integrate utility and primary or secondary reinforcers.

### Goal Value

The value of a goal is the internal representation of the expected reward or utility associated with the process or outcome of goal pursuit (e.g., can include utility that accrues during or after goal attainment) and drives the selection and initiation of goal-directed behaviors. The value associated with pursuing goals is likely comprised of both anticipatory and retrospective computations (Knutson et al., 2001; Levine et al., 2021; Van Boven & Ashworth, 2007). In other words, value can likely be attributed to goals in a process that draws from previous memories (Vaidya & Badre, 2020) and automatic schemas (Farovik et al., 2015; Santoro et al., 2016), or via a process of mental simulation (Kappes & Morewedge, 2016), like affective forecasting (T. D. Wilson & Gilbert, 2005) or aspiration (Kim & Ferrari, 2025). Crucially, these computations integrate both reward-seeking and punishment-avoidance motivational strategies, so that individuals may prioritize goals that either maximize gains or minimize losses, when these can’t be reconciled (Eldar et al., 2016; Sharp et al., 2022). Beyond these basic valence dimensions, value also reflects a complex interplay between pragmatic considerations—such as instrumental utility—and hedonic or intrinsic enjoyment. These components can be aligned, increasing likelihood of selecting a goal, or in conflict, reflecting a tension between extrinsic and intrinsic motivations that complicates goal-selection (Chu et al., 2024; Ryan & Deci, 2000).

### Goal Reachability

Goal reachability captures both (i) effort efficacy— the rate at which the subjective costs of mobilizing control to pursue a goal (which range in magnitude) can actually lead to progress towards a goal, and (ii) irreducible likelihood — the probability that the outcome will occur, independent of one’s effort exertion. Formal accounts in decision theory and computational control treat reachability as an expected-value computation in which prospective benefits are combined with both probability of success and effort costs to determine whether, where, and how strongly to engage control (Shenhav et al., 2013). For instance, the Expected Value of Control framework specifies that control is deployed when anticipated gains, weighted by efficacy (the probability that control will influence the outcome), exceed the costs of effort (Shenhav et al., 2013). This is demonstrated empirically at the behavioral and neurophysiological level, where orthogonal manipulations of reward and efficacy show that people invest more control when both the stakes and the likelihood that control “matters” are high (Frömer et al., 2021; Grahek et al., 2023). Complementing this, neuroimaging findings also reveal that cortico-insular and valuation networks continuously encode the subjective value of exerting effort to pursue a goal, providing evidence for the on-going cost-benefit evaluation of engaging in goal pursuit (Aridan et al., 2019; Arulpragasam et al., 2018).

When thinking about the irreducible likelihood of a goal occurring, regardless of effort exertion, goals that are highly likely to be reached are more often chosen than goals that are unlikely to be reached (Cohen & Jaffray, 1988). This probability estimation, however, can be miscalculated (discounted or inflated) by humans. For example, humans often overweight small probabilities and underweight moderate ones (Green & Myerson, 2004; Kahneman & Tversky, 1979; Tversky & Kahneman, 1992), sometimes leading to arguably sub-optimal goal-selections. Accordingly, and in line with the Motivational Intensity Theory (MIT), individuals are likely to work to pursue a goal so long as success remains sufficiently likely (considering both effort efficacy and irreducible likelihood) and “important” (highly valuable, as described above) (Brehm & Self, 1989). In other words, efforts towards even highly valuable goals will be abandoned if they are perceived to be unreachable.

### Goal Timeline

A third dimension that likely impacts goal selection is goal timeline: the temporal distance to anticipated costs and benefits. A large literature shows that people discount delayed outcomes (often hyperbolically), such that longer delays reduce subjective value (Frederick et al., 2002). Indeed, neuroimaging work indicates that valuation circuits (e.g., ventral striatum, medial prefrontal cortex) encode these delay-adjusted values during intertemporal choice (Frederick et al., 2002; Green & Myerson, 2004; Kable & Glimcher, 2007). Temporal distance also changes how options are mentally represented: as outcomes move further into the future, individuals rely on more abstract construals, which can shift preferences and prioritization of long- vs. near-term goals (Trope & Liberman, 2010). Clinically, populations with temporal estimation or time-preference disturbances illustrate why timeline matters for goal selection (Keidel et al., 2024). In attention-deficit/hyperactivity disorder, meta-analytic and experimental evidence shows broad time-perception deficits and delay aversion/steeper delay discounting, which bias choices toward immediate rewards and make distant goals harder to sustain without scaffolding (Jackson & MacKillop, 2016; Metcalfe et al., 2024; Van Dessel et al., 2018). Consistent with this, structured goal-management interventions can improve everyday functioning by externalizing timelines and progress cues (In De Braek et al., 2017). Similarly, in substance use disorder, robust meta-analyses and recent studies show elevated delay discounting, linking a shortened temporal horizon to selection of short-term, high-immediacy goals over longer-term objectives (e.g., health, employment), even when effort or success probability are unchanged (Amlung et al., 2017; Fontes et al., 2025; MacKillop et al., 2011). Together, these findings motivate treating goal timeline as a separable contributing dimension –alongside goal value and reachability– in models of goal selection.

## Towards a computational account of goal selection

Despite growing evidence that goals can directly alter lower-level RL processes, there has yet to emerge a consensus definition for the operationalization of a goal. Prior operationalizations include: a desired state (analogous to a goal state in goal-conditioned RL) (Botvinick et al., 2009; Cecchi et al., 2026; Schaul et al., 2015), a temporally extended sub-policy (analogous to an option (Sutton et al., 1999)), or something akin to a habit or policy-level attractor (Dolan & Dayan, 2013), a reward function (Dayan & Niv, 2008), or a task set (Collins & Frank, 2013; Sakai, 2008). Nevertheless, it is generally agreed that goals act to constrain which outcomes are valued (Molinaro & Collins, 2023b), which states and actions are relevant (Molinaro & Collins, 2023a), and what temporal horizon is prioritized (Green & Myerson, 2004). For the purposes of this article, we define goals as a latent, higher-order construct that determines which lower-level learning and decision-making processes are applied.

This formulation is consistent with other extensions of high-order accounts (Lieder & Griffiths, 2020). In other words, our proposal is best understood as an extension of, rather than a replacement for, reinforcement-learning accounts. In a standard RL task, the researcher typically fixes the state space, action set, reward function, and horizon. In everyday behavior, however, individuals often select or infer these elements for themselves. A participant who approaches a task with a loss-avoidance goal may assign subjective utility to preventing errors or avoiding embarrassment, whereas another participant may prioritize reward maximization. The same observed choices could therefore reflect differences in low-level learning parameters, differences in the subjective goal being pursued, or both. This is precisely where formal models can be useful: not only by estimating hidden processes, but also by revealing when a task is unable to distinguish between competing mechanistic explanations. This prediction is also consistent with recent empirical demonstrations that goal framing modulates RL parameters—including learning rates, inverse temperatures, and discount factors—across individuals (Frömer et al., 2025; Molinaro & Collins, 2023b).

The three dimensions emphasized above can each be mapped onto existing computational constructs. Goal value corresponds to the subjective utility or reward function attached to a process or outcome. Reachability corresponds to perceived efficacy, controllability, probability of success, and effort cost—constructs formalized in frameworks such as the Expected Value of Control (Shenhav et al., 2013) and in recent work linking goal attainability and effort allocation to behavior (Hall et al., 2024). Timeline corresponds to temporal discounting, planning horizon, and the delay between effort and expected reinforcement. Goal selection can then be modeled as latent arbitration over candidate goals, with behavior generated by the selected goal’s implied state representation, reward function, policy set, and stopping rule.

This framework generates clinically relevant predictions, some of which we have exemplified in detail below: internalizing symptoms may arise when individuals persistently select goals that are unreachable, organized around loss avoidance, excessively short-term, or drawn from an impoverished goal space. Such maladaptive goal representations can produce behavior resembling altered reward sensitivity, punishment sensitivity, or reduced effort allocation—even when basic outcome updating is intact. A hierarchical architecture also provides a principled account of how low-level deficits may give rise to goal-selection problems over time: a biased lower-level RL loop (e.g., an attentional bias toward threat) can progressively distort higher-level goal representations, shifting the distribution of selected goals without requiring separate higher-level dysfunction at onset.

## Viewing maladaptive behaviors through the lens of goal-selection

In this section, we illustrate how transdiagnostic symptoms in internalizing psychopathology may be conceptualized in the context of problematic goal-selection rather than low-level deficits. We will focus on providing a potential conceptual model for the onset and maintenance of repetitive negative thinking (RNT) and anhedonia, specifically (see Figure 2 for a visual aid). The following is supported by established empirical findings and insights from clinical practice.

**Figure 2:**
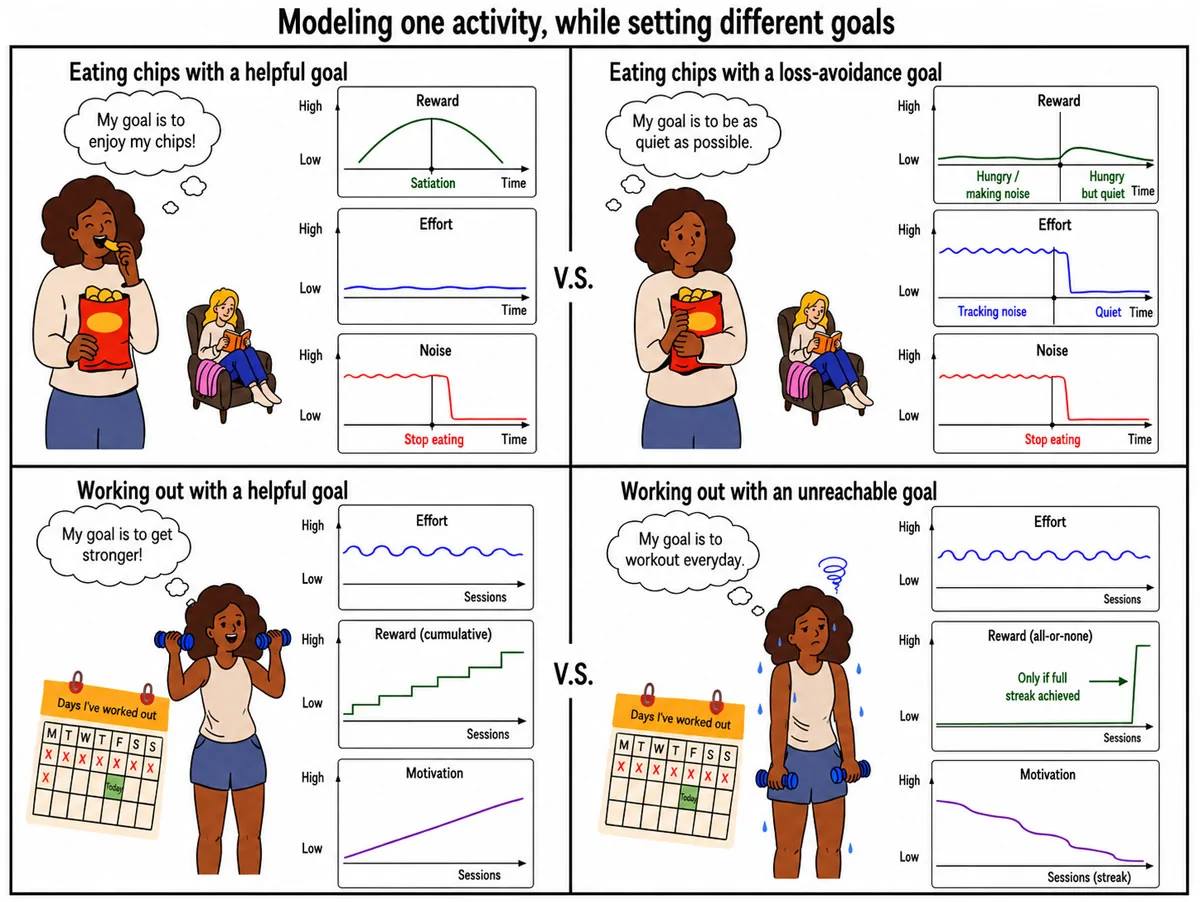
Visual representation of how goals shape activity perception. Top row: loss-avoidance goals can increase rumination and reduce pleasurable experiences. Bottom row: unreachable goals can increase rumination and decrease mood and motivation.

### Unreachable goals

People who engage in RNT sometimes report setting unreachable goals. For example, clinical accounts show patients endorsing a desire to reach full certainty over an event (Leahy, 2005). The first time that such an individual thinks about a past or future event, they may indeed derive some valuable insights. However, as this same person continues to revisit these topics to try and continue to progress towards their goal of reaching complete certainty, eventually they will misallocate mental effort because no more insight can be gained or uncertainty reduced. This is not to say that the act of thinking about negative past or future events can never be helpful. In fact, replaying events in our minds can serve as imaginal exposures and help with emotion processing and behavioral activation (Gros et al., 2012). The crux of the issue is the *goal with which this thinking is being done*. When the goal is unreachable, like gaining absolute certainty over the unknowable, control over the uncontrollable, or preventing a painful reality from having occurred, the thinking process becomes rigid, unproductive, and emotionally costly.

Similarly, setting overly ambitious goals that are *de facto* unreachable is also likely to keep individuals trapped in unreachable goal loops, prolonging distress and worsening mood. For example, individuals with strict and very high standards may set unrealistic goals, like going from not exercising at all to wanting to exercise every morning for 2 hours. This goal-setting style is likely to lead to a lot of disappointments when individuals, unavoidably, fail to reach these objectives. With time, as people repeatedly encounter situations in which their effort does not help them reach their goals, they may eventually learn to internalize a sense of hopelessness, triggering thoughts like “why should I work out at all? I can never keep to my routine”. Computationally, this may arise from an observation that progress towards the goal has halted (which must eventually happen when setting unreachable goals), leading to reduced opportunities to work towards/enjoy accomplishing sub-goals (Hall et al., 2024). Further, correctly observing that effort no longer serves to make progress towards the goal (or, observing that effort efficacy is low) should (Brehm & Self, 1989) lead to a reduced willingness to exert effort (Frömer et al., 2021; Grahek et al., 2023). Repeated experiences where effort fails to produce meaningful progress towards one or more goals may hence contribute to a broader reduction in motivation to engage in effortful processes entirely. This latter proposal aligns closely with the computational account of consummatory anhedonia proposed by Hall et al. (2024), which argues that pleasure is generated not merely by reward receipt but by perceived progress toward valued goals. Under this framework, diminished opportunities to register meaningful goal progress reduce consummatory pleasure. Our proposal extends this account to motivational anhedonia by suggesting that persistent selection of unreachable goals can itself create environments in which genuine progress is rarely experienced, thereby providing one mechanism through which reduced goal-progress signals and subsequent motivational deficits might arise. Ultimately, unreachable goal setting can lock individuals into RNT patterns that drain cognitive resources without resolution and, over time, may contribute to motivational anhedonia (Burns, 1980), as individuals lose confidence in the efficacy of their efforts and disengage from goal-directed behavior altogether.

This pattern may also constrain the set of goals that individuals generate or consider in the first place. When repeated effort has consistently failed to produce meaningful progress, individuals may cease to simulate or aspire toward rewarding future objectives (MacLeod & Salaminiou, 2001; Roepke & Seligman, 2016), resulting in an impoverished goal space before lower-level valuation processes even engage (K. S. Dickson et al., 2012; Hall et al., 2024)—another potential upstream contributor to motivational anhedonia. These dynamics may also operate differently across illness stages: early unreachable goal pursuit may drive the development of RNT and self-doubt; over time, it maintains avoidance and reduced contact with positive reinforcement; and as hopelessness generalizes across domains (Abramson et al., 1989), it may contribute to the progression of motivational deficits more broadly.

Note that most low-level processes must be intact for this “learned helplessness” to occur. Indeed, for this tracking of low efficacy to be possible, individuals must be aware of their performance, the expected consequences of their performance, the actual feedback that they receive, and the error between what they expected vs. observed to finally update efficacy estimates. Crucially, we propose that the deficit lies not in outcome tracking per se, but in failing to use those signals to revise the goal itself—a dissociation between prediction error encoding and goal-space updating. In other words, the lower-level RL system correctly registers that effort is not producing the expected outcome, yet the higher-level goal-selection process persists in selecting the same unreachable objective rather than recalibrating toward a more achievable one. It is this failure of goal revision in the face of accumulated negative prediction errors that we argue constitutes the core higher-order deficit.

### Loss-avoidance goals

Alternatively, some individuals may set goals based on fear of potential losses. The primary criterion for these goals is to choose activities that minimize the possibility of undesirable outcomes, whereas reward-seeking goals balance risk of loss with opportunities for gain. As with unreachable goals, emphasizing loss-avoidance goals over reward-seeking goals can also lead to RNT and anhedonic presentations. Indeed, some individuals set loss-avoidance goals as opposed to reward-pursuit goals as a form of self-protection from the negative affect associated with unwanted events. These individuals may, for example, repeatedly think about how poorly they performed in their first exam of the semester, rather than how well they did in their most recent exam, to keep expectations low. Their goal is to prevent experiencing disappointment when grades come out rather than celebrate a job well done in their second exam. As observed by a patient of Dr. Michelle Newman’s: “*[I prefer dreary weather over sunny days because] at least with rainy days you know what to expect; with sunny days you never know how long until the rain is ‘gonna’ come and snatch away any joy you might be having*.” (Newman et al., 2014) This type of goal setting nicely aligns with Dr. Newman’s empirically-supported contrast avoidance theory (Newman & Przeworski, 2024), which posits that worry is “*employed to prolong and maintain a negative emotional state thereby avoiding an unexpected negative emotional shift, or contrast experience*” (Newman & Llera, 2011).

Moreover, the tendency to focus on preventing losses (rather than seeking rewards) may also lead to reduced experiences of pleasure in daily life. In fact, the same activity can be experienced in a significantly less rewarding manner if loss-avoidance goals are set in place. For example, eating a bag of potato chips can be a highly rewarding experience, if one is focused on enjoying their taste, or it can be low reward if one is focused on being as quiet as possible to prevent bothering surrounding people with loud crunchy noises. When these loss-avoidance goals are sought after in more generalizable settings, these reductions in enjoyment can be experienced in a wider range of daily activities, potentially leading to stable reports of consummatory anhedonia. Imagine someone who goes about their week with the goal of “not embarrassing themselves in front of their peers”. This person is likely to derive less pleasure from conversations at work, texting, social media scrolling, etc. than if their goal was “connecting with others”.

Crucially, this maladaptive goal setting may not be captured in, for instance, simple lab-based gambling tasks measuring low-level processes. Individuals who set loss-avoidance goals in their daily life need not lack the ability to learn from win and loss trials, nor may they be more or less sensitive to the value of monetary/point losses or wins. Instead, their reward functions and state spaces may look drastically different than the researcher’s model expectations.

We note that individuals setting loss-avoidance goals may not experience pleasure as long as they maintain these goals but may preserve the *capacity* to experience pleasure. Accordingly, this framework relates to self-reported experiences of consummatory anhedonia rather than potential biological deficits to derive pleasure from even unexpected rewards, despite these often being undifferentiable. Across illness stages, loss-avoidance goal framing may initially develop as a targeted cognitive self-protective response, then maintain consummatory anhedonia by consistently reducing in-the-moment reward contact across daily activities and ultimately generalize to broad patterns of engagement.

## Goal understanding, identification, and selection are crucial in therapeutic environments

Various highly effective psychotherapies assume that lower-level processes are either intact or able to resolve themselves without direct intervention. Accordingly, they seek to use those low-level mechanisms to target higher-order cognitive and behavioral change. Here, we describe the general function of goal setting in the context of several “gold-standard”, evidence-based psychotherapies.

Goal setting is often considered a crucial component at the onset of any therapeutic relationship (Michalak & Holtforth, 2006). Goal setting has proven helpful in promoting patient motivation and compliance (Bassett & Petrie, 1999), and providing more opportunities for behavioral reinforcement during progress tracking (Brunstein, 1993; Grosse & Grawe, 2002). Further, goal setting has been shown to improve therapist-client alignment (Angeli et al., 2019; Renger & Macaskill, 2021), as well as help patients direct attention towards behaviors/thoughts of interest and dismiss irrelevant content (Wollburg & Braukhaus, 2010).

Notably, even beyond this initial goal setting procedure, goal identification and pursual have also become core components of many empirically supported psychotherapies for internalizing psychopathology (Cuijpers et al., 2007; Gloster et al., 2020; Hofmann et al., 2012). For example, Acceptance and Commitment Therapy focuses on helping patients select values-aligned goals and activities (to increase self-actualization and agency) rather than behaviors that will exclusively reduce distress in the short-term (which perpetuate avoidance and prevent new learning from happening) (Chase et al., 2013; Hayes & Pierson, 2005). Notice that this intervention heavily relies on functional low-level perceptual, attentional, reward sensitivity, and reinforcement learning systems to effectively shape behavior and improve mood. The proposed mechanism of action of this intervention, consistent with its theoretical model, is to assist patients in reshaping their perceived goal-space, so new, often more rewarding options feel available to them again. Patients clearly present with the ability to encode punishments (aversive outcomes), but they must conserve the ability to valuate and encode rewards/reinforcers as well, even while experiencing unpleasant emotions, for these interventions to prove helpful in reducing symptoms. The importance of a patient’s goal space is further highlighted by supplemental interventions, like Motivational Interviewing. This person-centered strategy simply brings attention to a patient’s ambivalence and helps them clarify their goals to themselves. This single-session intervention proves effective in helping patients sustain engagement in psychotherapy and behaviorally activate (Lundahl et al., 2010; Rubak et al., 2005).

Cognitive Behavioral Therapy then takes goal setting to the next level by providing psychoeducation on “SMART” goals. This acronym helps patients learn that goals ought to be: Specific, Measurable, Achievable, Realistic, and Time-Limited (Stewart et al., 2024). Similarly, Behavioral Activation therapy helps individuals identify “mastery” versus “pleasure” tasks (tasks that bring one closer to long-term goals of “proficiency” vs. “enjoyment”) so that they may structure their days to both restore function and affect (Furukawa et al., 2018). These psychotherapies also assume working low-level processes like cost-reward computations, decision-making, and executive function. Interestingly, these psychotherapies also work to reshape a patient’s goal space, but via a more experiential approach. Rather than reminding patients of their values and the reasons why certain choices are important to them, these cognitive-behavioral interventions rely on individuals completing rewarding activities to learn new habits, decide on more adaptive goals, and eventually restore positive mood. In other words, if a patient presented with an inability to learn from rewards and punishments, perform cost-reward computations, and action plan and execute, these psychotherapies would not be fruitful. Together, goal understanding, identification, and selection are fundamental pieces in multiple evidence-based practices that further assume intact low-level cognitive processes to help address internalizing pathology problems. For a comprehensive list of the roles of goals and low-level cognition in various psychotherapies, see Table 1.

**Table 1:** Summary of evidence-based therapies and their relation to low- and high-level cognitive processes.


EVIDENCE-BASED THERAPY		CAUSE OF ILLNESS	ROLE OF PATIENT GOALS	STATE OF LOWER-LEVEL PROCESSES	FUNCTIONING LOW-LEVEL PROCESSES	POTENTIAL GOAL BIAS

**Cognitive Behavioral Therapy (Hollon & Beck, 1994)**	CBT	Unhelpful ways of thinking and learned unhelpful behaviors.	Patients set SMART goals to pursue over the course of treatment.	Possibly intact.	Operant and instrumental learning processes are assumed intact and used to help patients acquire more adaptive behaviors.	Loss-avoidance, short-term goals, or unreachable goals.

**Cognitive Processing Therapy (Greenberg, 1995)**	CPT	Unhelpful generalized thoughts about the self or the world.	Goals are discussed in the context of seeking motivation for change.	Attention and memory retrieval are thought biased towards negative events.	All learning systems, however, are assumed intact, so individuals can gather and incorporate evidence to challenge their unhelpful beliefs.	Loss-avoidance over reward-seeking goals.

**Exposure and Response Prevention (Rowa et al., 2007)**	ERP	Engaging in unhelpful learned behaviors to reduce distress.	Goals are set (collaboratively) between sessions for patients to engage in exposures on their own time.	Attention is believed biased towards threatening stimuli.	Inhibitory and reinforcement learning processes are assumed intact to diminish compulsive behavior.	Loss-avoidance or short-term goals

**Prolonged Exposure (Foa, 2011)**	PE	Unhelpful beliefs and avoidance of stimuli that trigger distress.	Goals are set to increase willingness and compliance. Between sessions, patients also set exposure goals to complete on their own.	Attention is believed biased towards threatening stimuli.	Habituation and inhibitory learning processes are assumed intact and used to reduce emotional reactions to traumatic events.	Loss-avoidance over reward-seeking goals.

**Behavioral Activation (Cuijpers et al., 2007)**	BA	Reduction in behaviors that provide a sense of pleasure and mastery.	Patients set daily goals to complete activities aimed at increasing their sense of “pleasure” and “mastery”.	Possibly intact.	All perceptual, attentional, and learning processes are assumed intact for this psychotherapy to prove effective.	Unreachable goals.

**Acceptance and Commitment Therapy (Hayes & Pierson, 2005)**	ACT	Intolerance of distressing emotions.	Patients select values-aligned goals and tasks over distress-avoidance ones, to reach self-actualization.	Attention and memory retrieval are thought to be biased towards negative events.	All other reward/punishment valuation and learning systems are assumed intact.	Loss-avoidance over reward-seeking goals.

**Dialectical Behavioral Therapy (Linehan, 1987)**	DBT	A lack of emotion-regulation and social skills.	DBT diary cards track progress towards personal goals. Emphasis is put on skills that will help patients maintain a “life worth living”, often the central patient goal.	Natural evidence processing is considered impaired, so that covert learning processes have failed.	Overt learning processes are utilized to develop adaptive skills.	Unreachable goals or short-term goals.

**Interpersonal Psychotherapy (Weissman, 1994)**	IPT	Unstable, distant, or explosive social relations.	Treatment is focused on problem-solving ways to change target relationships towards the patient’s desired goals.	Social habits are assumed unhelpful.	Learning processes, goal-directed decision-making, and executive functioning are assumed intact.	Excessively short-term or loss-avoidance or unreachable goals.

**Eye Movement Desensitization and Reprocessing (D. L. Wilson et al., 1996)**	EMDR	Excessive emotional arousal related to experienced trauma.	Goals are discussed in the context of seeking motivation for change.	Attention and memory retrieval are believed biased towards threatening stimuli.	Inhibitory learning processes are assumed intact and used to reduce emotional reactions to traumatic events.	Loss-avoidance over reward-seeking goals.

**Mindfulness-Based Cognitive Therapy (Sipe & Eisendrath, 2012)**	MBCT	Unhelpful ways of thinking.	Patients select daily goals to complete activities aimed at increasing their sense of “pleasure” and “mastery”.	Perceptual and attentional systems are believed to be biased towards adverse stimuli.	Perceptual and attentional systems are also assumed flexible and malleable using mindfulness.	Loss-avoidance, short-term goals, or unreachable goals.


In line with our core argument that internalizing psychopathology is heterogeneous and may emerge from high- as well as low-level deficits, we must acknowledge that psychotherapy interventions targeting lower-level processes can also prove effective for some psychopathologies. Indeed, exposure therapies for often chronic conditions like Prolonged Exposure (Foa, 2011) (for Post-Traumatic Stress Disorder) and Exposure and Response Prevention (Rowa et al., 2007) (for Obsessive Compulsive Disorder) are great examples of the power of habituation, and inhibitory learning, respectively, (both low-level cognitive processes) in effectively treating anxiety-related mental illnesses centered around an over-active sympathetic nervous system, and attentional and memory retrieval biases. These interventions, notably, do not aim to directly perform any skills training or cognitive restructuring, proving most helpful for anxiety or fear-conditioned illnesses (Powers et al., 2010; Rowa et al., 2007). Individuals with comorbid disorders may need concurrent treatment to address co-existing conditions (Gordon et al., 2023; Van Minnen et al., 2012). This reality provides further support for our claim that there is a place for research trying to understand the role of low-level cognition on the onset and maintenance of psychopathology, alongside higher-order cognition.

## Concluding remarks and future perspectives

Taken together, the success of these empirically validated interventions highlights the disconnect between targets of psychotherapeutic intervention and current computational psychiatry research. Indeed, only a minority of computational psychiatry studies have relied on established psychotherapy frameworks to guide hypothesis selection (see Norbury et al., 2024, for a recent example). We believe that a greater emphasis should be placed on examination of deficits in the types of higher-order processes, like goal-selection, that are often the focus of successful interventions. This is not a call to abandon research on lower-level processes, which are also important targets for some psychotherapeutic interventions. However, for many psychotherapies there remains a gap between the formal models tested in computational psychiatry research and clinical practice. To close this gap, we encourage the field to pursue task designs and models that allow participants to set, revise, and pursue a wider variety of goals—not just single, experimenter-defined targets. Such efforts will facilitate our ability to separate low vs. high level impairments. Furthermore, flexible paradigms that vary goal attributes (e.g., value, reachability, timeline, social stakes) and allow participants to define their own goals based on those features would likely improve ecological validity and maybe increase finding replicability rates. In the present work, we have described how these different attributes may reveal distinct cognitive-pathophysiologies within goal-selection.

Recent findings on free action planning show that people can spontaneously generate and evaluate action sequences without explicit instruction, underscoring the feasibility of using open-ended, self-initiated tasks that capture how goals are selected and updated in the wild (Ho et al., 2022; Ten et al., 2021). This redirection could even draw from research exploring effort allocation and effort-based decision making in and out of the lab setting. For example, recent work has suggested that reduced willingness to expend effort may critically depend on whether the goal is to obtain reward or avoid punishment (Bustamante et al., 2024; Senta et al., 2025). Alternatively, ecological assessment paradigms may allow for greater participant-defined goal-pursuit (Cooper et al., 2025). Option generation tasks may also reveal great insight into impoverished or biased processes in psychopathology, and thus we recommend that option generation be measured and modeled directly, when possible, in future research (Kalis et al., 2013; Morris et al., 2021). Finally, because clinical decision-making unfolds under uncertainty, partially observable formulations that infer latent beliefs and goals from sparse, noisy evidence may be powerful tools to identify patient-specific misbeliefs and cognitive deficits (Albarracin & Pitliya, 2022; Croom et al., 2023). At the very least, accounting for self-reported participant goals to customize task instructions or stimuli may be helpful, and collecting participant reports or ratings on task attitudes and goals (as much as insight into such constructs is possible in lab tasks) may help identify confounding variables or serve as a quality check.

This proposed reorientation also generates some testable empirical predictions to assess whether goal-selection deficits are separable from, and clinically meaningful beyond, the low-level process failures that have been the primary focus of the field to date.

- First, goal-selection deficits should be partially dissociable from basic RL deficits: individuals with intact trial-by-trial outcome learning but impaired goal revision should show inconsistent behavior across task contexts that vary goal framing, rather than uniformly altered learning rates.
- Second, if variability in goal selection can drive symptom heterogeneity, then individual differences in pre-task goal endorsement (e.g., whether a participant adopts a loss-avoidance versus reward-seeking orientation) should predict systematic variance in fitted RL parameters, and their interpretation—such as asymmetric learning rates or reduced sensitivity to positive outcomes—over and above diagnostic status alone.
- Third, experimentally manipulating goal framing within a task (e.g., instructing participants to “avoid losing” vs. “earn as much as possible”) should shift RL outputs in predictable, dissociable ways in clinical versus healthy groups.
- Fourth, ecological momentary assessment measures of naturalistic goal quality (such as perceived reachability and value alignment) should mediate the relationship between psychopathology severity and effort allocation outcomes.

In sum, we advocate for an extension or re-orientation towards tasks that enable participant-defined goal pursuit and models that infer goals, beliefs, policies, and stopping rules. Such models would allow computational psychiatry to test whether behavior that appears to reflect altered reward learning, punishment sensitivity, effort allocation, or exploration instead reflects differences in the higher-order goal being pursued. The purpose of this re-orientation is to unearth better markers of, and mechanisms underlying, internalizing psychopathology while preserving the strengths of existing lower-level computational approaches.

## Outstanding questions box

1. Do individuals have conscious insight into the goals they set?
2. How can computational and clinical models deal with competing goals?
3. Can we observe domain-specific vs. domain-general deficits in goal selection?
4. How can computational psychiatrists and intervention scientists productively collaborate to develop testable hypotheses around mechanisms of goal-selection?
5. Do externally vs. internally generated goals differentially impact overt and covert behavior?
