# Supplementary material for: What’s the Goal? Reconsidering the Importance of Higher-Order Cognition in Internalizing Psychopathology
Source: Comput Psychiatr. 2026 Nov 13;10(1):237–54. doi: 10.5334/cpsy.184 (PMC13761889; doi:10.5334/cpsy.184)
Supplement: Peer review history

# Reviewer report: Round 1, Reviewer A

**DOI:** 10.5334/cpsy.184.r1

## Recommendation:

Accept with Major Revision

## Comments/Explanation (required). Please include references to any minor or major revisions required, as well as addressing any issues with language clarity. Authors will see these comments:

The paper argues that computational psychiatry has overly focused on low-level cognitive processes (e.g., reinforcement learning, attention biases) and proposes that higher-order processes, specifically goal selection, may better explain internalising psychopathology. The authors discuss evidence from psychotherapeutic interventions and reinforcement learning studies and focus on three key dimensions of goal selection: value, reachability, and timeline. Finally, they use the examples of repetitive negative thinking (RNT) and anhedonia to illustrate how internalising psychopathology may be conceptualised as maladaptive goal-selection, and the role of goal setting in current psychotherapies. They conclude by calling for more self-initiated, goal-flexible task paradigms to better align computational psychiatry with clinical practice.

Overall, the core argument is compelling, important and thoroughly put. The disconnect between psychotherapeutic interventions and computational methods is an important issue and the consideration of higher-level processes to bridge this divide is a useful insight.

However, the paper currently reads as a perspective, and presents limited computational formalisation beyond a conceptual framework for a Computational Psychiatry submission.

Main points

– The construct of ‘goal selection’ and its discussed dimensions of goal value, reachability and timeline remain conceptual without any discussion of how they may be formalised or how they map on to a computational framework. The authors might want to consider recent work by Hall, Huys and Browning.
– What is a ‘goal’ computationally? If using an RL framework, is this a goal state or a policy or something else? Or is this situated within a non-RL framework?
– These components could be a reframing of existing RL concepts, or the authors may be suggesting a completely new formulation. If it is a reframing, what does this new computational framework add beyond the existing models?
– There is conceptual discussion of low-level processes ‘evolving’ into higher-order processes – what is the computational framework for this process?
– How does the broad importance of goals speak to other evidence-based therapies - if this is not the case, how should the relevance to depression / anxiety be conceptualized?

Minor points:

– There are a few strong assumptions which do not appear to be empirically supported – i.e. that ‘low-level processes are frequently intact’ (pg. 3), the ‘mechanism of action’ and patients’ preserved ‘ability to encode negative reinforcers’ in ACT (pg. 13)
– There are several descriptions of how goals lead to symptoms - but many of these lack citations. I wonder whether insights from the author’s clinical practice should be labelled as such.
– Manuscript states it will discuss goal-selection with respect to RNT and anhedonia, but there is a substantial and developed discussion of RNT with very brief and somewhat underdeveloped mention of anhedonia
– Manuscript states goal-selection deficits could explain development, maintenance, and/or progression of internalising psychopathology but there is no explicit mention of these distinct aspects later in the paper
– The manuscript presents an interesting discussion but has some limited empirical utility - How would these ideas actually be tested, or what evidence would support this reconceptualisation? Are there any empirical predictions?
– The figures are very visually appealing, but they do not add so much. It would be useful to have some more computational/empirical visualisations. The table contains a lot of densely presented non-essential information.

# Reviewer report: Round 1, Reviewer D

**DOI:** 10.5334/cpsy.184.r2

## Recommendation:

Accept with Minor Revision

## Comments/Explanation (required). Please include references to any minor or major revisions required, as well as addressing any issues with language clarity. Authors will see these comments:

Review of ‘What’s the goal? Reconsidering the importance of higher-order cognition in internalizing psychopathology’

I very much enjoyed reading this article. I found the core argument compelling: many evidence-based psychotherapies are founded on the hypothesis that, in most people struggling with internalizing disorders, basic low-level processes related to attention, reinforcement learning (RL), and memory are intact, they simply need to be re-directed such that the individual will pursue better goals that will increase their sense of mastery and pleasure, rather than reinforcing avoidance, repetitive negative thinking, learned helplessness, and so on. If the low-level processes were not intact then these therapies would not work, which calls into question our field’s heavy focus on low-level processes. Of course, as the authors know, the therapies do not work well for many people, which implies that the low-level processes really are off in some individuals. Nevertheless, the basic point stands and I agree with the call for more work on participant-driven goal pursuit. I think the article would be of interest to readers of Computational Psychiatry, and I found the point made in the first paragraph on page 5 (continued from page 4) particularly compelling—namely, failing to account for variability participant’s goals can negatively affect virtually all aspects of RL research. I did not have any great concerns while reading, but below I offer major and minor comments, which I hope may help the authors strengthen an already strong paper.

Major comments:

1. The authors are clear throughout that by directing attention to the issue of higher-order goals, they are not arguing that research on lower-level processes be abandoned. Nevertheless, they motivate the article by stating that the main goal of prior work on low-level processes (including attention, reinforcement learning [RL], and memory) has been to develop generative models whereby dysfunction in such processes gives rises to symptomatic behavior, and they argue that inconsistent findings (e.g., sometimes depression negatively affects RL, sometimes it doesn’t), calls the value of this endeavor into question. I basically agree but raise two points that may merit comment. First, if we stay in this framework (studying low-level processes to explain/understand higher-order problems), we still must be cognizant that the large gap in levels of analysis ensures no one-to-one correspondence. To continue with the same example, a diagnosis of Major Depressive Disorder (MDD) is reasonably coherent at the symptom level but almost certainly implicates a wide range of lower-level mechanisms, such that we cannot expect every individual with MDD to have a deficit in RL. A reasonable rejoinder is to ask, well then, what percentage of people with MDD should we expect to have such problems? I don’t know the answer, but I think the point still stands: we need to set reasonable expectations such that we do not throw out the baby with the bathwater. Second, it can be useful to ‘flip the script’, by including participants with psychopathology in studies of lower-level processes less to explain the psychopathology and more to see how those low-level processes can break down. In other words, do not study RL to ‘explain’ MDD, but include adults with MDD in RL studies because some subset of them are likely to exhibit weak prediction errors, blunted response biases, poor effort expenditure, etc., and by studying those failures we can learn more about RL (or attention or memory or whatever). And to link this to the first point, if our understanding of the low-level processes improves sufficiently, then we should be able to offer a clear explanation of breakdowns in low-level processes at the individual-level, which would let us dispense with group-level inference and get us around the wide gap between diagnoses and basic processes mentioned earlier. To summarize, I think I see the same problem as the authors but would suggest a complementary solution: study the basic mechanisms in adults with internalizing disorders, expect dysfunction in a small-ish percentage of the sample, and work to get to a point where that dysfunction can be identified and characterized sufficiently well that it has diagnostic value for the people in whom it is present.
2. I was compelled by the discussion of overambitious goals leading to repeated failure leading to motivational anhedonia; that account makes sense to me. However, I am not sure I entirely agree with this: ‘Note that most low-level processes must be intact for this ‘learned helplessness’ to occur. Indeed, for this tracking of low efficacy to be possible, individuals must be aware of their performance, the expected consequences of their performance, the actual feedback that they receive, and the error between what they expected vs. observed to finally update efficacy estimates.’ (p. 10) If the system was working as it should, then negative prediction errors should drive down value estimates such that the individual would learn to set more reasonable value estimates going forward (i.e., having failed to work out for two hours each morning last January, this New Year’s I will set a more sensible goal, like walking to work). As a complementary example, it is common for depressed adults to be unmoved by a positive prediction error that would delight non-depressed people, and—because value estimates seem not to be updated properly—to go into subsequent, similar states still expecting negative outcomes, despite having experienced a positive prediction error recently.
3. One big challenge: it is hard to bring open-ended goal-setting under laboratory control in a way that does not destroy ecological validity—the goals that people are willing to endorse in a lab setting may be different from those they hold when not in the lab, and it may take time and experience to learn that a particular goal is faulty, which (again) makes it hard to study in the lab. An obvious solution is to ditch the lab and study these things with smartphones or some similar technology, but typically studies that use such approaches lose contact with the lower-level mechanisms mentioned throughout the article. If the authors have more concrete ideas about how a ‘participant-defined goal pursuit’ study could be designed to avoid or address these concerns, such material would be a welcome addition.

Minor

1. I was confused by this sentence, on the top of page 7: ‘In other words, efforts towards even highly valuable goals will not be abandoned if they are perceived to be unreachable.’ Should that be ‘will be abandoned’? Or maybe ‘unreachable’ should be ‘reachable’ and ‘even’ should be deleted? Please clarify.
2. On page 13, the manuscript mentions ‘negative reinforcers’ but I think the authors roughly mean ‘punishments’ here, not things that, when removed, lead to relief or other positive emotions, right? Consider rephrasing.
3. Page 16: ‘For example, recent work has suggested that reduced willingness to avoid effort may critically depend ‘; I wondered if ‘avoid’ should be ‘expend’ here?
4. In the Outstanding Questions box, I found ‘consciously aware insight’ a bit awkward. There are also some minor typos throughout the manuscript that should be corrected (e.g., in the last Outstanding Question, ‘differentially impact over and mental behavior’).

# Reviewer report: Round 2, Reviewer A

**DOI:** 10.5334/cpsy.184.r3

## Recommendation:

Accept (No Revisions Necessary)

## Comments/Explanation (required). Please include references to any minor or major revisions required, as well as addressing any issues with language clarity. Authors will see these comments:

Thank you for the thoughtful responses. All our comments were fully addressed.

## Consent to Publish

Consent Given

# Reviewer report: Round 2, Reviewer B

**DOI:** 10.5334/cpsy.184.r4

## Recommendation:

Accept (No Revisions Necessary)

## Comments/Explanation (required). Please include references to any minor or major revisions required, as well as addressing any issues with language clarity. Authors will see these comments:

Review of ‘What’s the goal? Reconsidering the importance of higher-order cognition in internalizing psychopathology’ (R1) The authors were responsive to my comments and the revised manuscript is an improvement. I think the new section—‘Towards a computational account of goal selection’—is helpful, and while I agree with the other reviewer that the manuscript reads mainly as a ‘perspective’, I think it is a helpful perspective and a useful take on extensive research on reinforcement learning in relationship to psychopathology. Below I list a few last minor comments that the authors can likely handle without need for additional review.

Minor comments:

1. There is a typo on page 4, ‘aid reach more ecologically valid’.
2. On page 6—‘goals that are highly probable to be reached are more often chosen than goals that are unlikely to be reached’; consider replacing ‘probable’ with ‘likely’. In the next sentence, ‘differentially’ can be cut.
3. Regarding the first bullet point on page 19, as the authors know a lower learning rate is not always bad—in a stable environment, a low learning rate is often helpful. Consequently, consider revising ‘uniformly depressed learning rates’.
4. Regarding the third bullet point on page 19, about instructing participants to avoid losing vs. maximizing their winnings: the authors may be interested in studies by Ian Dobbins and colleagues that manipulate criterion settings in recognition memory experiments—i.e., participants are incentivized to maximize Hits (which also increases False Alarm rates) in some blocks, whereas they are incentivized to maximize Correct Rejections (which reduces the Hit and False Alarm rate) in other blocks. These studies seem to exemplify the goal setting perspective the authors are describing, albeit in a different setting (recognition memory in healthy students vs. reward/punishment processing in clinical groups). It is interesting that this approach is readily explained by signal detection theory: it corresponds to a shift in response bias, with no change in discriminability. Although there are reward-related paradigms that assess the impact of internalizing on response bias vs. discriminability, my goal here is only to alert the authors to another line of work that may be relevant—I don’t see a need for edits along these lines.

## Consent to Publish

Consent Given

# Reviewer report: Round 1, Author Response

**DOI:** 10.5334/cpsy.184.r5

**Response to Reviewers**

We are grateful to both reviewers for their thoughtful and constructive comments. Both reviewers identified a central issue in the previous version of the manuscript: although the conceptual argument for goal selection was clear, its computational status and empirical implications required greater specification. In response, we have substantially revised the manuscript to clarify what we mean by a ‘goal’ computationally, to specify how goal value, reachability, and timeline can be formalized within existing computational frameworks, and to articulate what this higher-order framework adds beyond standard reinforcement-learning accounts. We have also added a new section outlining concrete empirical predictions and task designs, and revised the discussion of low-level processes to avoid overstatement. All edits are highlighted in blue.

Reviewer 1

## Reviewer A:

**Major Comment 1:** *The manuscript reads as a perspective and presents limited computational formalization for a Computational Psychiatry submission*.

## Author’s response

**Response:** We agree that this is fundamentally a perspective and offers a conceptual blueprint for creating a formal or simulation-based computational account rather than directly proposing a computational account itself. We recognize that this may fall short of the reviewer’s critique. Nevertheless, we believe the points raised in this piece are sufficiently relevant to the readership of Computational Psychiatry to warrant publication in this journal.

In our revision, we have taken steps to expand the computational description of goals and goal-selection and offered a fuller articulation of how we conceptualize goals: as latent higher-order control representations (g) that determine the computational problem to which lower-level learning and decision-making processes are applied. We added a new section titled “Toward a computational account of goal selection”.

## Reviewer A:

**Major Comment 2:** *The construct of goal selection and the dimensions of value, reachability, and timeline remain conceptual and should be mapped onto a computational framework, including recent work by Hall, Huys, and Browning*.

## Author’s response

**Response:** We have added an explicit mapping between goal-selection dimensions and computational constructs. Goal value is now compared to a subjective utility or reward function; reachability to perceived efficacy, controllability, probability of success, and effort cost; and timeline to temporal discounting, planning horizon, and delay to reinforcement.

See Page 9: “The three dimensions emphasized above can each be mapped onto existing computational constructs. Goal value corresponds to the subjective utility or reward function attached to a process or outcome. Reachability corresponds to perceived efficacy, controllability, probability of success, and effort cost—constructs formalized in frameworks such as the Expected Value of Control and in recent work linking goal attainability and effort allocation to behavior. Timeline corresponds to temporal discounting, planning horizon, and the delay between effort and expected reinforcement. Goal selection can then be modeled as latent arbitration over candidate goals, with behavior generated by the selected goal’s implied state representation, reward function, policy set, and stopping rule.”

We added references to Hall, Huys, and Browning’s work on anhedonia potentially resulting from a deficit in goal-progress tracking or sub-goal achievement. Our proposed presentation of “unreachable goals” builds off of these ideas, and we now make this connection more explicit.

See Page 11: “This latter proposal aligns closely with the computational account of consummatory anhedonia proposed by Hall, Browning, and Huys’, which argues that pleasure is generated not merely by reward receipt but by perceived progress toward valued goals. Under this framework, diminished opportunities to register meaningful goal progress reduce consummatory pleasure. Our proposal extends this account to motivational anhedonia by suggesting that persistent selection of unreachable goals can itself create environments in which genuine progress is rarely experienced, thereby providing one mechanism through which reduced goal-progress signals and subsequent motivational deficits might arise. ”

## Reviewer A:

**Major Comment 3:** *What is a ‘goal’ computationally? If using an RL framework, is this a goal state or a policy or something else? Or is this situated within a non-RL framework?*

## Author’s response

**Response:** We have now sought to more explicitly address this question. We define a goal as a latent higher-order control representation, g, that constrains which outcomes are valued, which states and actions are relevant, and what temporal horizon is prioritized—and thus determines the computational problem to which lower-level learning and decision-making processes are applied. Under this account, a goal may be operationalized as a desired state (goal-conditioned RL), a temporally extended sub-policy (an option), a policy-level attractor, a reward function, or a task set, depending on the experimental context.

See Page 8-9: “Despite growing evidence that goals can directly alter lower-level RL processes, there has yet to emerge a consensus definition for the operationalization of a goal. Prior operationalizations include: a desired state (analogous to a goal state in goal-conditioned RL), a temporally extended subpolicy (analogous to an option), or something akin to a habit or policy-level attractor, a reward function, or a task set. Nevertheless, it is generally agreed that goals act to constrain which outcomes are valued, which states and actions are relevant, and what temporal horizon is prioritized. For the purposes of this article, we define goals as latent, higher-order construct that determines which lower-level learning and decision-making processes are applied.”

## Reviewer A:

**Major Comment 4:** *These components could be a reframing of existing RL concepts, or the authors may be suggesting a completely new formulation. If it is a reframing, what does this new computational framework add beyond the existing models?*

## Author’s response

**Response:** We now clarify that the framework is an extension of RL rather than a replacement for it. However, please note that we are not offering a full computational framework in the present paper – our scope is narrower in nature, and it aims to propose a new conceptual framework to spark future computational and empirical work.

See Page 8-9: “This formulation is consistent with other extensions of high-order accounts. In other words, our proposal is best understood as an extension of, rather than a replacement for, reinforcement-learning accounts. In a standard RL task, the researcher typically fixes the state space, action set, reward function, and horizon. In everyday behavior, however, individuals often select or infer these elements for themselves. A participant who approaches a task with a loss-avoidance goal may assign subjective utility to preventing errors or avoiding embarrassment, whereas another participant may prioritize reward maximization. The same observed choices could therefore reflect differences in low-level learning parameters, differences in the subjective goal being pursued, or both. This is precisely where formal models can be useful: not only by estimating hidden processes, but also by revealing when a task is unable to distinguish between competing mechanistic explanations. This prediction is also consistent with recent empirical demonstrations that goal framing modulates RL parameters—including learning rates, inverse temperatures, and discount factors—across individuals.”

## Reviewer A:

**Major Comment 5:** *There is conceptual discussion of low-level processes ‘evolving’ into higher-order processes – what is the computational framework for this process?*

## Author’s response

**Response:** We appreciate this observation. We agree this is an important point, but believe it is outside the manuscripts core focus. We now mention this more explicitly as an important avenue for future work:

“Moreover, it is also possible that what may have started as a low-level process deficit may have evolved to a goal-selection deficit by the time individuals reach a researcher’s testing room or a therapy session. Consider, for example, a learned attentional bias towards threatening stimuli, following a traumatic event.”

## Reviewer A:

**Major Comment 6:** *How does the broad importance of goals speak to other evidence-based therapies - if this is not the case, how should the relevance to depression/anxiety be conceptualized?*

## Author’s response

**Response:** We have now clarified that goals need not be the crux of illness across all internalizing psychopathology, nor do they need to be targeted to effectively treat mental illnesses. Rather we now further clarify that some anxiety-related illnesses can be effectively treated using exposure-based therapies.

See Page 16: “In line with our core argument that internalizing psychopathology is heterogeneous and may emerge from high, as well as low, level deficits, we must acknowledge that psychotherapy interventions targeting lower-level processes can also prove effective for some psychopathologies. Indeed, exposure therapies for often chronic conditions like Prolonged Exposure (for Post-Traumatic Stress Disorder) and Exposure and Response Prevention (for Obsessive Compulsive Disorder) are great examples of the power of habituation, and inhibitory learning, respectively, (both low-level cognitive processes) in effectively treating anxiety-related mental illnesses centered around an overactive sympathetic nervous system, and attentional and memory retrieval biases.”

## Reviewer A:

**Minor Comment 1:** *There are a few strong assumptions which do not appear to be empirically supported – i.e. that “low-level processes are frequently intact” (pg. 3), the “mechanism of action” and patients’ preserved “ability to encode negative reinforcers” in ACT (pg. 13)*

## Author’s response

**Response:** As written, we agree that the paper implied unsupported assumptions and have made targeted revisions throughout. We have qualified the language throughout, removing all overconfident causal claims.

## Reviewer A:

**Minor Comment 2:** *There are several descriptions of how goals lead to symptoms - but many of these lack citations. I wonder whether insights from the author’s clinical practice should be labelled as such*.

## Author’s response

**Response:** We revised the language throughout to distinguish clinical insights from established empirical findings. Further, we have added additional references to clinical accounts by Drs. Newman, Leahy, and Seligman.

## Reviewer A:

**Minor Comment 3:** *Manuscript states it will discuss goal-selection with respect to RNT and anhedonia, but there is a substantial and developed discussion of RNT with very brief and somewhat underdeveloped mention of anhedonia*

## Author’s response

**Response:** We addressed this by expanding the anhedonia discussion within existing sections. In ‘Unreachable goals,’ we further developed the discussion of motivational anhedonia. In ‘Loss-avoidance goals,’ we added language on how consummatory anhedonia may be developed and maintained.

## Reviewer A:

**Minor Comment 4:** *Manuscript states goal-selection deficits could explain development, maintenance, and/or progression of internalizing psychopathology but there is no explicit mention of these distinct aspects later in the paper*

## Author’s response

**Response:** Rather than adding a dedicated subsection, we integrated development/maintenance/progression signposting into the existing sections. The ‘Unreachable goals’ section now links unreachable goal pursuit to symptom development (via RNT and self-doubt), maintenance (via avoidance and disengagement), and progression (as hopelessness generalizes across domains). The ‘Loss-avoidance goals’ section similarly traces the illness stage trajectory of loss-avoidance framing from onset through maintenance and progression.

See Page 11: “This pattern may also constrain the set of goals that individuals generate or consider in the first place. When repeated effort has consistently failed to produce meaningful progress, individuals may cease to simulate or aspire toward rewarding future objectives, resulting in an impoverished goal space before lower-level valuation processes even engage—another potential upstream contributor to motivational anhedonia. These dynamics may also operate differently across illness stages: early unreachable goal pursuit may drive the development of RNT and self-doubt; over time, it maintains avoidance and reduced contact with positive reinforcement; and as hopelessness generalizes across domains, it may contribute to the progression of motivational deficits more broadly.”

And See Page 13: “Across illness stages, loss-avoidance goal framing may initially develop as a targeted cognitive self-protective response, then maintain consummatory anhedonia by consistently reducing in-the-moment reward contact across daily activities, and ultimately generalize to broad patterns of engagement.”

## Reviewer A:

**Minor Comment 5:** *The manuscript presents an interesting discussion but has some limited empirical utility - How would these ideas actually be tested, or what evidence would support this reconceptualization? Are there any empirical predictions?*

## Author’s response

**Response:** We added a new paragraph of empirical predictions to the concluding section. Specifically, we propose: (1) individual differences in pre-task goal endorsement should predict systematic variance in fitted RL parameters over and above diagnostic status; (2) experimentally manipulating goal framing should shift RL outputs in predictable, dissociable ways; (3) goal-selection deficits should be partially dissociable from basic RL deficits; and (4) EMA measures of naturalistic goal quality should mediate the relationship between psychopathology severity and effort allocation outcomes. Please see Page 19.

## Reviewer A:

**Minor Comment 6:** *The figures are very visually appealing, but they do not add so much. It would be useful to have some more computational/empirical visualizations. The table contains a lot of densely presented non-essential information*.

## Author’s response

**Response:** We have now revised Figure 2 to include a more computational visualization or our proposed framework. We have also edited the existing table for conciseness, but have retained its comprehensive mapping of evidence-based treatments to goal types.

## Reviewer D:

Reviewer 2

**Major Comment 1:** *[…] I […] raise two points that may merit comment. First… we still must be cognizant that the large gap in levels of analysis ensures no one-to-one correspondence. To continue with the same example, a diagnosis of Major Depressive Disorder (MDD) is reasonably coherent at the symptom level but almost certainly implicates a wide range of lower-level mechanisms, such that we cannot expect every individual with MDD to have a deficit in RL. A reasonable rejoinder is to ask… what percentage of people with MDD should we expect to have such problems? […]Second, it can be useful to “flip the script”… by including participants with psychopathology in studies of lower-level processes less to explain the psychopathology and more to see how those low-level processes can break down. In other words, do not study RL to “explain” MDD, but include adults with MDD in RL studies because some subset… are likely to exhibit weak prediction errors, blunted response biases, poor effort expenditure, etc., and by studying those failures we can learn more about RL… And to link this to the first point, if our understanding of the low-level processes improves sufficiently, then we should be able to offer a clear explanation of breakdowns in low-level processes at the individual-level, which would let us dispense with group-level inference and get us around the wide gap between diagnoses and basic processes mentioned earlier. To summarize… I see the same problem as the authors but would suggest a complementary solution: study the basic mechanisms in adults with internalizing disorders, expect dysfunction in a small-ish percentage of the sample, and work to get to a point where that dysfunction can be identified and characterized sufficiently well that it has diagnostic value for the people in whom it is present*.

## Author’s response

**Response:** We appreciate this point and agree. We revised the introduction and conclusion to clarify that our goal-selection account is complementary to low-level computational approaches, not a replacement. We now more explicitly state that low-level learning, valuation, attention, and memory mechanisms may be primary for some individuals or subgroups, and that studying these processes in internalizing samples remains valuable for identifying individual-level mechanisms. Our clarified framing is that goal selection may explain some inconsistencies in group-level findings, while leaving intact the research program of characterizing low-level processes and their clinical relevance in specific individuals.

See Page 3: “Complementing these heterogeneous results from laboratory studies is the evidence from psychotherapy intervention studies, which suggest that low-level processes may be intact for some patients.”

See Page 4: “Our claim is not that low-level cognitive processes never play a causal role in the development of internalizing psychopathology (indeed, see Goal understanding, identification, and selection are crucial in therapeutic environments for a discussion of effective psychotherapies that target low-level deficits). Rather, we suggest that this approach may fail to capture causal mechanisms for individuals who primarily struggle with higher-order processes.”

See Page 16: “In line with our core argument that internalizing psychopathology is heterogeneous and may emerge from high, as well as low, level deficits, we must acknowledge that psychotherapy interventions targeting lower-level processes can also prove effective for some psychopathologies.”

## Reviewer D:

**Major Comment 2:** *I was compelled by the discussion of overambitious goals leading to repeated failure leading to motivational anhedonia… However, I am not sure I entirely agree with this: “Note that most low-level processes must be intact for this “learned helplessness” to occur. Indeed, for this tracking of low efficacy to be possible, individuals must be aware of their performance, the expected consequences of their performance, the actual feedback that they receive, and the error between what they expected vs. observed to finally update efficacy estimates.” (p. 10) If the system was working as it should, then negative prediction errors should drive down value estimates such that the individual would learn to set more reasonable value estimates going forward (i.e., having failed to work out for two hours each morning last January, this New Year’s I will set a more sensible goal, like walking to work). …it is common for depressed adults to be unmoved by a positive prediction error that would delight non-depressed people, and—because value estimates seem not to be updated properly—to go into subsequent, similar states still expecting negative outcomes, despite having experienced a positive prediction error recently*.

## Author’s response

**Response:** We agree with this point and have revised the relevant paragraph substantially. We now distinguish between detecting low efficacy (which may be supported by intact lower-level updating) and adaptively revising the goal itself (which represents the proposed higher-order deficit). The revised account proposes that individuals may encode repeated failure sufficiently to update global effort beliefs while failing to flexibly revise unrealistic goal conditions, or overgeneralizing from local failure. We also note that unreachable goal pursuit may interact with lower-level learning alterations, including impaired updating from positive outcomes. The key dissociation is between prediction error encoding and goal-space revision.

See Page 12: “Crucially, we propose that the deficit lies not in outcome tracking per se, but in failing to use those signals to revise the goal itself—a dissociation between prediction error encoding and goal-space updating. In other words, the lower-level RL system correctly registers that effort is not producing the expected outcome, yet the higher-level goal-selection process persists in selecting the same unreachable objective rather than recalibrating toward a more achievable one. It is this failure of goal revision in the face of accumulated negative prediction errors that we argue constitutes the core higher-order deficit.”

## Reviewer D:

**Major Comment 3:** *One big challenge: it is hard to bring open-ended goal-setting under laboratory control in a way that does not destroy ecological validity—the goals that people are willing to endorse in a lab setting may be different from those they hold when not in the lab, and it may take time and experience to learn that a particular goal is faulty, which… makes it hard to study in the lab. An obvious solution is to ditch the lab and study these things with smartphones or some similar technology, but typically studies that use such approaches lose contact with the lower-level mechanisms mentioned throughout the article. If the authors have more concrete ideas about how a “participant-defined goal pursuit” study could be designed to avoid or address these concerns, such material would be a welcome addition*.

## Author’s response

**Response:** This is an excellent point, and pose a real challenge for laboratory studies. We have expanded our concluding section to include more options for participant-defined goal-pursuit studies. Specifically, we elaborate on the potential of EMA paradigms and highlight that self-reported task goals could be either used to better craft task instructions or stimuli, or, at the very least, collected to gauge data quality concerns.

See Page 18: “Alternatively, ecological assessment paradigms may allow for greater participant-defined goal-pursuit.”

See Page 19: “At the very least, accounting for self-reported participant goals to customize task instructions or stimuli may be helpful, and collecting participant reports or ratings on task attitudes and goals (as much as insight into such constructs is possible in lab tasks) may help identify confounding variables or serve as a quality check.”

## Reviewer D:

**Minor Comment 1:** *Page 7: ‘efforts towards even highly valuable goals will not be abandoned if they are perceived to be unreachable’ -- should ‘not’ be removed?*

## Author’s response

**Response:** Thank you - this was a typo.

## Reviewer D:

**Minor Comment 2:** *Page 13: ‘negative reinforcers’ should probably be ‘punishments’ or ‘aversive outcomes.’*

## Author’s response

**Response:** Corrected. We replaced this with “punishments (aversive outcomes)”.

## Reviewer D:

**Minor Comment 3:** *Page 16: ‘reduced willingness to avoid effort’ should be ‘reduced willingness to expend effort.’*

## Author’s response

**Response:** Corrected.

## Reviewer D:

**Minor Comment 4:** *‘Consciously aware insight’ in the Outstanding Questions box is awkward; there are also typos (‘goals-selection’, ‘over and mental behavior’)*.

## Author’s response

**Response:** All corrected.
